# Adaptation of the “Language Use Inventory” to Brazilian Portuguese

**DOI:** 10.1590/2317-1782/e20240177en

**Published:** 2025-07-28

**Authors:** Beatriz Servilha Brocchi, Jacy Perissinoto, Ellen Osborn

**Affiliations:** 1 Faculdade de Fonoaudiologia, Pontifícia Universidade Católica de Campinas – PUC-Campinas - Campinas (SP), Brasil.; 2 Departamento de Fonoaudiologia, Universidade Federal de São Paulo – UNIFESP - São Paulo (SP), Brasil.

**Keywords:** Language Development, Child Language, Parental Relationship, Preschool, Evaluation Study

## Abstract

**Purpose:**

To adapt the “Language Use Inventory” parent -report measure for Brazilian Portuguese children.

**Methods:**

A total of 254 Brazilian parents and children participated in the survey, comprising five groups within an age range from 18 to 47 months. The translated and pre-adapted version of the Language Use Inventory (LUI) measure into Brazilian Portuguese was used in online and in person format. Analysis of Cronbach’s alpha coefficients was used to verify the internal reliability, and Kruskal-Wallis and Mann-Whitney tests were used to analyze the form of completion, sex, child’s age group, and parents’ education level.

**Results:**

Comparative analyses between the completion methods did not identify significant differences between the printed and online formats. The Brazilian Portuguese version of the LUI demonstrated high internal consistency with respect to all subscales and with respect to the entire measure (α>0.99). Girls obtained higher scores than boys for Part 2 (p<0.001) and Part 3 (p=0.001) and on the Total LUI score (p=0.001). Children whose parents had more years of schooling obtained higher scores in Parts 2 and 3.

**Conclusion:**

The similarity of results related to internal reliability and developmental trends of the Brazilian Portuguese version of LUI as compared with the original English version, supports its use as a reliable instrument to assess pragmatic language functioning in Brazilian preschoolers.

## INTRODUCTION

Pragmatic ability is reliant on different social knowledge bases and cognitive systems of interaction, which are considered the most complex aspects of linguistic functionality^(1,2)^. The acquisition and development of human language skills are socio-biological processes involving human sociocognitive skills of understanding and sharing intentionality^(3)^. Thus, understanding communicative intent is necessary for language development^(4)^.

Interest in the set of skills to use language appropriately in the context of social interaction has been highlighted with respect to the development of research in language area^(1,5)^. These could provide to researchers and practitioners evaluative processes and measures that can achieve greater accuracy.

Those researches could also assist in more accurate diagnoses and guide early interventions and developmental monitoring. Furthermore, would support programs and supportive strategies for parents and caregivers^(1-3,6)^.

### Development of the pragmatics of language

We adopt the perspective that since six months of age, the child already understands that people have goals; at nine months, the child understands the interlocutor’s action and reacts accordingly (e.g., the adult gives him/her a toy) and can differentiate goals, actions, and perceptions coming from the others’ behavior; and from 12 to 18 months, the child is able to follow what the adult points to in a referential situation and infers the reasons for the others’ communicative behavior. Children aged 18 months can interpret an adult’s social goals differently for the same communicative act, trying to reproduce what the adult is trying to do (and not what they did)^(7)^.

From the age of two years, children learn turn-taking, engage in greater conversational topic initiation, adaptation of their utterances to conversational participants’ knowledge states, and the production of longer utterances and stories^(8)^. They use language for a broad range of functions, including to request, inform, ask, joke, and comment. They can initiate and maintain dialogue for several turns, and converse with people in concrete and familiar contexts. Schulze and Tomasello^(7)^ argue that at 26 months, children can understand communicative intentions, and based on this recognition, infer the social intention of the interlocutor. Between three and four years, communicative functions are refined and intensified, for example, to seek missing factual information with questions. Children’s utterances also become much more intelligible and coherent^(9)^.

According to several authors, alterations in the functionality of using linguistic components in different communicative contexts pragmatics of language can have repercussions on social interaction, behavior, self-esteem, and academic skills^(10-12)^. The resulting difficulties are consistently associated with Autism Spectrum Disorders (ASD) and can be implicated in other neurodevelopmental disorders, such as Developmental Language Disorder (DLD)^(13)^, Attention Deficit Hyperactivity Disorder (ADHD), conduct disorders, and oppositional defiant disorder^(14)^.

Thus, the importance of pragmatic skills for successful communication has been recognized by efforts to design and evaluate assessment instruments^(1,13,15)^. Russell and Grizzle^(14)^ emphasize the need to assess, as early as possible, the content, structural/dimensional, ecological, and diagnostic validity of instruments assessing pragmatic skills, such as diagnostic tests, behavioral checklists/questionnaires, and structured participant observations.

With regard to the pragmatics of language, there are few standardized and validated instruments that verify its development before the age of 4^(8)^. There are many protocols but not pragmatics directly. Regarding protocols for observing the development of pragmatics, the Communication and Symbolic Behavior Scale test (CSBS) is a standardized and validated protocol designed to evaluate preschool children who are at risk for communication disorders. The Children's Communication Checklist protocol was developed and validated to access information on aspects of communication disorders, about pragmatic changes, social communication and qualitative aspects of speech and language. The protocol consists of questions aimed at caregivers of children over 4 years old.

In relation to national protocols, the ABFW pragmatics test (child language test that assesses the development of aspects of phonology, vocabulary, fluency and pragmatics) analyzes the functional aspects of communication through observation of free play therapist/ caregiver and child, verifying the communicative acts performed between them during the interaction, the means used and the communicative functions. Another Brazilian instrument is the Behavioral Observation Protocol (PROC)^(9)^ with the aim of understanding the typical evolution of language development, symbolism and the relationship between such aspects of development, but mainly, it makes it possible to configure the levels evolution and modes of cognitive and communicative functioning presented by children complaining of developmental delays or disorders.

The instruments described above are widely used in Brazil, but there is no standardized protocol in the national literature that evaluates the development of pragmatics in preschool children, observing the use of language in everyday life, from the perspective of parents at early ages, especially up to 4 years of age.

Thus, the authors sought to develop a Brazilian Portuguese version of an established and empirically validated parent-report measure, the Language Use inventory (LUI)^(16)^.

### Language use inventory

The LUI^(16)^ is a Canadian instrument developed and initially validated by O’Neill^(8)^ and Pesco and O’Neill^(17)^. It consists of a standardized and validated parent-report measure designed to assess the development of pragmatics in children aged 18 to 47 months. The LUI focuses on the child’s use of language in everyday life situations with various interlocutors and for a broad range of purposes and capitalizes on parents’ uniquely extensive knowledge of these areas of language use of their child that would be very difficult to assess by an examiner.

The LUI is composed of 14 subscales organized into three parts. Part 1, “How does your child communicate through gestures,” has two subscales: A-“How your child uses gestures to ask for something” (with 11 items) and B-“How your child uses gestures to make you notice something,” (with 2 items.) Part 2 is titled “Your child’s communication through words” and has three subscales: C-“Type of words your child uses” (with 21 items); D-“Your child’s requests for help” (7 items); and E-“Your child’s interests (5 items – not scored).” Parents are directed to continue to complete Part 2 if their child has started using at least one word regularly. Part 3, “Your child’s phrases,” has 9 subscales: F-“How your child uses words to make you notice something” (6 items); G-“Questions and comments from your child” (9 items); H- “Questions and comments from your child about yourself and other people” (36 items); I-“Your child’s use of words in activities with others” (14 items); J-“Your child’s teasing and sense of humor” (5 closed and 1 open item); “K-Your child’s interest in words and language” (12 items); L-“Your child’s interests when speaking” (2 closed and 5 open items – not scored); M-“How your child adapts conversation with others” (15 items); and N-“How your child constructs sentences and stories: (36 items).

The LUI includes a LUI Score Sheet to tally the scores to determine the child’s raw scores for its scored subscales, Parts and LUI Total score. The LUI Total score is obtained by summing the scores from the 10 scored subscales within Parts 2 and 3 and thus reflects a measure of spoken language only as it does not include the two subscales pertaining to gesture use in Part 1.

The LUI has been used in several countries in North America, Europe and Asia to assess pragmatic language development^(17,18)^ and in studies with clinical populations such as autistic children^(19,20)^, and children with ADHD^(21)^, with cerebral palsy^(22)^, and Down Syndrome^(23-25)^. It has been used to assess pragmatics in neglected children as well^(26,27)^.

It has been translated and validated for use in other languages such as European Portuguese^(28)^, Canadian French^(29)^, Polish^(30)^, Norwegian^(31)^, Chinese^(32)^, and Italian^(33)^.

### Pilot study of the LUI-Portuguese (Brazil)

In Brazil, the LUI has been translated and a pilot study was conducted to test its reliability^(34)^. The authors aimed to translate and adapt the LUI from English into Brazilian Portuguese. This study was conducted in two stages. Following permission granted by the publisher of the LUI, the process of translation and back-translation of the protocol began, adapting it to sociocultural aspects such as expressions, names, and examples in Brazilian Portuguese. Analysis of Cronbach’s alpha coefficients was used to assess the internal reliability of the translation process.

In the second stage of the pilot study, the translated version of the LUI was completed by 52 parents of children aged 18-47 months attending early childhood education schools in a city in the interior of São Paulo State. The results were analyzed with respect to the subscales of the questionnaire and LUI Total score. Moreover, the results were analyzed with respect to child age and parents’ educational levels. Cronbach’s alpha values revealed alpha values exceeding .88 for almost all subscales, except for B, D and J (.74-.77). The LUI Total score demonstrated high internal consistency with α > 0.98.

Thus, in this preliminary study, the authors observed that the Brazilian Portuguese version of the LUI demonstrated strong evidence of internal reliability and developmental trends with age similar to the results of the original English version of LUI, supporting its potential as a new measure to evaluate pragmatics of Brazilian children. The present study aims to adapt the LUI into Brazilian Portuguese. The potential role of additional variables such as child’s age, sex, and demographic variables was also investigated.

## METHODS

This prospective study was approved by the Research Ethics Committees from Pontifícia Universidade Católica de Campinas and Universidade Federal de São Paulo (no. 5.184.555/2019). All participants were evaluated from February 2020 to December 2022.

### Participants

A total of 274 parents or guardians of children (137 boys and 137 girls, aged 18-47 months) participated in this study. The children were grouped by age into five groups as follows: G1: 18-24 months; G2: 25-30 months; G3: 31-36 months; G4: 37-42 months; G5: 43-47 months (Table 1).

**Table 1:** Number and percentage of children by age group

Groups	Number	%
1	74	29.1
2	63	24.8
3	49	19.3
4	32	12.6
5	36	14.2
Total	254	100

**Figure 1:**
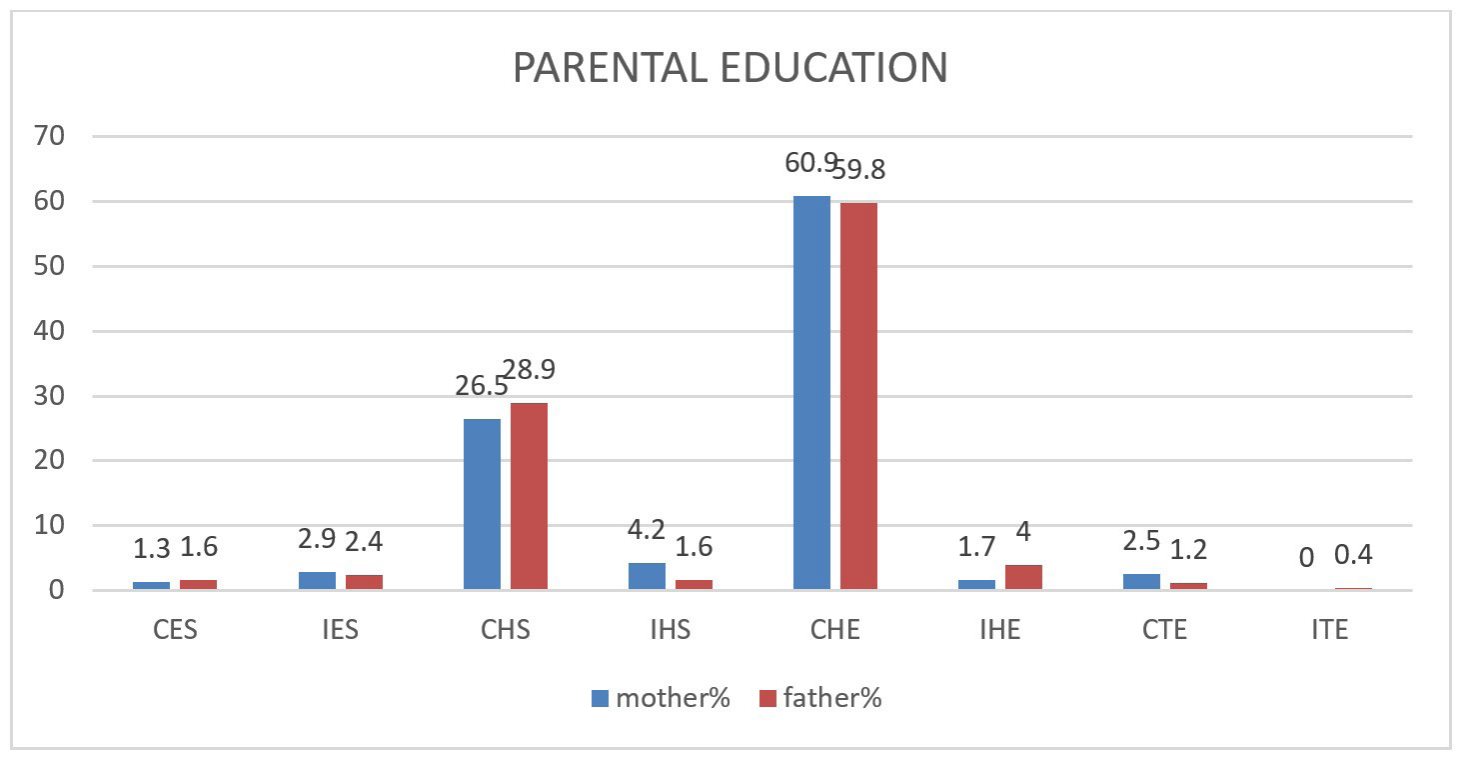
Maternal and paternal education of the research participants

**Figure 2:**
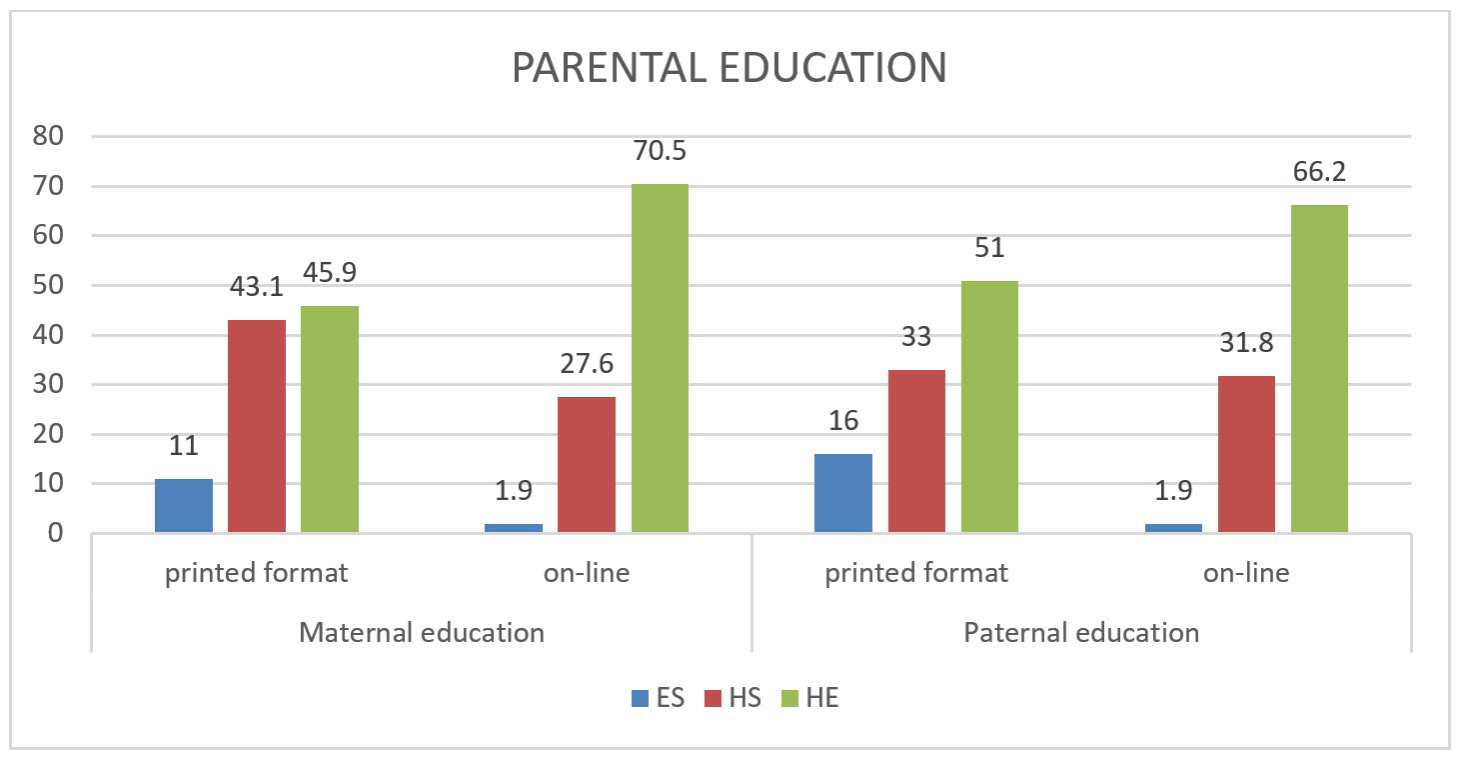
Comparison between schooling of parents who participated in the online and printed format

It was observed that more than half of the mothers (59.8%) and fathers (60.9%) had been to college, and 28.9% of mothers and 26.5% of fathers had completed high school, as shown in Figures 1 and 2. Almost all the questionnaires were completed by mothers (96.5%).

Caption: CES = complete elementary school; IES = incomplete elementary school; CHS = complete high school; IHS = incomplete high school; CHE = complete higher education; IHE = incomplete higher education; CTE = complete technical education; ITE = incomplete technical education

Caption: ES = elementary school; HS = high school; HE = higher education

All parents whose children were within the age range stipulated for the study (18-47 months), who signed the Informed Consent Form, and who completed the entire LUI questionnaire, were included.

Of the 281 LUI questionnaires collected, six were excluded from the study. Three of them were incomplete, two children presented syndromic conditions and one presented delayed neuropsychomotor development under investigation (described by their parents in the last part of the LUI), and was also excluded from the research.

### Procedure

The Language Use Inventory questionnaire translated into Brazilian Portuguese^(34)^, was used in the printed version and, with authorization from the author of the instrument, the online version. The sample was constituted through dissemination of the research and active search by the main researcher via social media, in person at childcare and volunteer services.

For both situations, after invitation and acceptance from the families, the online or in-person consent form was signed and the LUI was completed by the parents. The questionnaire was sent to the parents (individual link) or, in the in-person format, it was given to the parents for individual completion. The first page of the questionnaire contains an instruction manual for parents to complete. The researchers were available for any questions (in both formats).

The full completion depended on the age of the child and took between 10 and 30 minutes. 161 online questionnaires and 93 in-person questionnaires were fully completed.

Questions demand answers using one of the five alternatives (never, rarely, sometimes, always and not any more) matching the response formats on the original English LUI.

### Results analyses

The LUI Total test score was obtained by scoring each of the 10 scored subscales of Parts 2 and 3 comprising this score, as described in the LUI Manual accompanying the English version^(16)^. For dichotomous yes/no questions, YES answers receive 1 point. For the few questions with the four options never, rarely, sometimes or often, 0 points are assigned to never or rarely and 1 point to sometimes or often.

Initially, a comparison was made between the findings from the two different administration formats (in person versus online) to verify possible differences in the parents’ responses. We used the McNemar, Chi-square, and Mann-Whitney tests, considering a significance level of 0.05%. Regarding each subscale of the LUI, there were similarities in the answers between the two groups, except for subscales B (p<0.001), D (p=0.0049), and M (p=0.030), in which the online format had a higher average answer than the printed format. Given the similarity of the responses obtained in both formats, the data were grouped to verify the internal consistency of the instrument.

To assess internal reliability of the questions (items) within each subscale, we used Cronbach’s alpha (Values close to 1 indicate good internal consistency). For an exploratory survey, values above 0.6 were accepted. The results were also analyzed regarding to the variables of sex, age group of the child, and educational level of the parents. Descriptive percentages and statistical tests were used, such as Spearman’s correlation, Kruskal-Wallis, Tukey’s multiple comparisons, and Mann-Whitney tests, considering a significance level of 0.05%.

## RESULTS

### Internal reliability of LUI

Internal reliability was assessed for all subscales and the three parts of the LUI. As shown in Table 2, Cronbach’s alpha analysis showed excellent reliability for all three parts of the LUI, each with high internal consistency, and the LUI Total score, with α>0.99 (Table 2).

**Table 2:** Reliability of the parts that make up the surveyed instrument

	Subscale	Questions Number	Cronbach Alpha
Part 1	A	11	0.884
	B	2	0.738
	Total	13	0.873
Part 2	C	21	0.952
	D	7	0.772
	Total	28	0.951
Part 3	F	6	0.883
	G	9	0.947
	H	36	0.980
	I	14	0.871
	J	5	0.773
	K	12	0.888
	M	15	0.913
	N	36	0.966
	Total	133	0.989
Part 2 + 3	Total	161	0.990

Separate analysis of each subscale established that all but B showed high internal consistency (α between 0.75 and 0.96). The latter showed the lowest coefficient but was considered to have good internal consistency (α=0.73).

Children’s scores on LUI subscales were found to be related using Spearman’s correlations (Table 3).

**Table 3:** Relationship between the subscales from LUI parental Inventory

n=270		Total A	Total B	Part 1 (total)	Total C	Total D	Part 2 (total)	Total F	Total G	Total H	Total I	Total J	Total K	Total M	Total N	Part 3 (total)
TOTAL B	Correlation Coefficient	**0.144**														
	Sig. (p)	**0.018** *														
PART 1 (total)	Correlation Coefficient	**0.988**	**0.273**													
	Sig. (p)	<0.001*	<0.001*													
TOTAL C	Correlation Coefficient	**-0.578**	**-0.151**	**-0.592**												
	Sig. (p)	<0.001*	0.013*	<0.001*												
TOTAL D	Correlation Coefficient	**-0.333**	-0.098	**-0.346**	0.654											
	Sig. (p)	<0.001*	0.109	<0.001*	<0.001*											
PART 2 (Total)	Correlation Coefficient	**-0.536**	**-0.144**	**-0.551**	**0.960**	**0.781**										
	Sig. (p)	<0.001*	0.018*	<0.001*	<0.001*	<0.001*										
TOTAL F	Correlation Coefficient	**-0.485**	-0.092	**-0.491**	**0.800**	**0.643**	**0.793**									
	Sig. (p)	<0.001*	0.131	<0.001*	<0.001*	<0.001*	<0.001*									
TOTAL G	Correlation Coefficient	**-0.561**	-0.078	**-0.565**	**0.845**	**0.627**	**0.835**	**0.797**								
	Sig. (p)	<0.001*	0.204	<0.001*	<0.001*	<0.001*	<0.001*	<0.001*								
TOTAL H	Correlation Coefficient	**-0.480**	-0.014	**-0.468**	**0.734**	**0.527**	**0.716**	**0.711**	**0.813**							
	Sig. (p)	<0.001*	0.817	<0.001*	<0.001*	<0.001*	<0.001*	<0.001*	<0.001*							
TOTAL I	Correlation Coefficient	**-0.529**	**-0.125**	**-0.535**	**0.776**	**0.607**	**0.769**	**0.802**	**0.830**	**0.795**						
	Sig. (p)	<0.001*	0.040*	<0.001*	<0.001*	<0.001*	<0.001*	<0.001*	<0.001*	<0.001*						
TOTAL J	Correlation Coefficient	**-0.349**	-0.097	**-0.356**	**0.551**	**0.485**	**0.556**	**0.586**	**0.649**	**0.583**	**0.658**					
	Sig. (p)	<0.001*	0.111	<0.001*	<0.001*	<0.001*	<0.001*	<0.001*	<0.001*	<0.001*	<0.001*					
TOTAL K	Correlation Coefficient	**-0.495**	-0.044	**-0.495**	**0.750**	**0.540**	**0.728**	**0.725**	**0.845**	**0.796**	**0.820**	**0.668**				
	Sig. (p)	<0.001*	0.470	<0.001*	<0.001*	<0.001*	<0.001*	<0.001*	<0.001*	<0.001*	<0.001*	<0.001*				
TOTAL M	Correlation Coefficient	**-0.514**	-0.069	**-0.514**	**0.761**	**0.577**	**0.744**	**0.766**	**0.844**	**0.782**	**0.854**	**0.676**	**0.864**			
	Sig. (p)	<0.001*	0.260	<0.001*	<0.001*	<0.001*	<0.001*	<0.001*	<0.001*	<0.001*	<0.001*	<0.001*	<0.001*			
TOTAL N	Correlation Coefficient	**-0.559**	-0.092	**-0.560**	**0.802**	**0.592**	**0.785**	**0.786**	**0.883**	**0.837**	**0.878**	**0.668**	**0.871**	**0.901**		
	Sig. (p)	<0.001*	0.130	<0.001*	<0.001*	<0.001*	<0.001*	<0.001*	<0.001*	<0.001*	<0.001*	<0.001*	<0.001*	<0.001*		
PART 3- LUI (TOTAL)	Correlation Coefficient	**-0.555**	-0.074	**-0.552**	**0.815**	**0.603**	**0.797**	**0.816**	**0.906**	**0.924**	**0.912**	**0.706**	**0.910**	**0.923**	**0.963**	
	Sig. (p)	<0.001*	0.225	<0.001*	<0.001*	<0.001*	<0.001*	<0.001*	<0.001*	<0.001*	<0.001*	<0.001*	<0.001*	<0.001*	<0,001*	
total LUI	Correlation Coefficient	**-0.563**	-0.081	**-0.562**	**0.835**	**0.624**	**0.821**	**0.821**	**0.909**	**0.920**	**0.910**	**0.703**	**0.908**	**0.919**	**0.964**	**0.998**
	Sig. (p)	<0.001*	0.185	**<0.001**	<0.001*	<0.001*	<0.001*	<0.001*	<0.001*	<0.001*	<0.001*	<0.001*	<0.001*	<0.001*	<0.001*	<0.001*

* Significative correlation

Caption: Sig = significative

Significance was observed for almost all the 2 × 2 correlations. It is noteworthy that the positive correlation between the subscales of Part 1, referring to the use of gestures, and the negative correlation between these and the components of Parts 2 and 3; i.e., the use of gestures, is due to the decreased use of gestures as the use of words and sentences increases. Subscale B showed few significant relationships that were considered weak, even when they were significant (scores on B has so little variability, especially with only 2 items and children, even using words and phrases, continue using gestures. Scores in all 10 scored subscales in Parts 2 and 3 comprising the Total LUI Score were significantly related to each other, suggesting that the production of words, sentences, and narratives are related to each other.

After analyzing the internal aspects of LUI, the inventory was related to sex, children’s age, and parents’ education. With respect to gender, it was found that, from Part 1, both groups showed a similar decrease with increasing age. However, for all subscales that make up Parts 2 and 3, girls performed significantly better than boys; i.e., girls performance was higher with respect to the expressive subscales in Parts 2 and 3 (Table 4).

**Table 4:** Relationship between the scores obtained, according to the gender of the participants

		Male	Female	Mann-Whitney Test (p)
TOTAL A	Average	6.72	6.45	
	Median	7.00	7.00	0.398
	Standard deviation	3.73	3.42	
TOTAL B	Average	2.02	1.94	
	Median	2.00	2.00	0.336
	Standard deviation	0.87	0.76	
PART 1 (total)	Average	8.74	8.39	
	Median	10.00	9.00	0.268
	Standard deviation	4.05	3.72	
TOTAL C	Average	16.17	18.61	
	Median	20.00	21.00	0.001*
	Standard deviation	6.09	4.48	
TOTAL D	Average	5.75	6.46	
	Median	7.00	7.00	0.005*
	Standard deviation	2.19	1.54	
PART 2 (Total)	Average	21.91	25.07	
	Median	26.00	28.00	<0.001*
	Standard deviation	7.84	5.61	
TOTAL F	Average	3.91	4.74	
	Median	5.00	6.00	<0.001*
	Standard deviation	2.16	1.92	
TOTAL G	Average	5.08	6.65	
	Median	6.00	8.00	0.001*
	Standard deviation	3.72	3.23	
TOTAL H	Average	18.60	22.94	
	Median	19.00	28.00	0.007*
	Standard deviation	13.30	12.73	
TOTAL I	Average	7.57	9.46	
	Median	8.00	12.00	0.005*
	Standard deviation	5.31	5.53	
TOTAL J	Average	1.46	1.97	
	Median	1.00	2.00	0.012*
	Standard deviation	1.65	1.69	
TOTAL K	Average	5.19	6.88	
	Median	5.00	7.00	<0.001*
	Standard deviation	3.81	3.57	
TOTAL M	Average	6.79	8.80	
	Median	6.00	10.00	0.002*
	Standard deviation	5.05	4.82	
TOTAL N	Average	11.22	16.33	
	Median	8.00	17.00	<0.001*
	Standard deviation	10.97	11.44	
PART 3- (TOTAL)	Average	59.82	77.79	
	Median	55.00	92.00	0.001*
	Standard deviation	42.47	40.38	
TOTAL LUI	Average	81.73	102.86	
	Median	80.00	120.00	0.001*
	Standard deviation	48.98	44.76	

* Significative correlation

Regarding the effect of age on children’s scores, the Kruskal-Wallis test revealed that the youngest children used significantly more gestures than older children (p<0.001), as can be seen in the Total of Part 1 in which groups G1 and G2 used significantly more gestures than the other groups, especially the older ones.

The opposite can be observed when analyzing Part 2 (Table 5). The youngest group G1 used significantly fewer words than the next group (p<0.001) and the others (as can be seen in the subscales C and D that make up that part).

**Table 5:** Relationship between the age of the participants (divided into groups according to age group) and the response obtained in the inventory

			Group by age (months)								(p)		Results
			18 to 24 (1)		25 to 30 (2)		31 to 36 (3)		37 to 42 (4)	43 to 47 (5)			
TOTAL A	Average		8.77		6.70		5.92		4.91	4.28			
	Median		10.00		7.00		6.00		5.00	5.00	<0.001*		(1) > (2) = (3) = (4) = (5)
	Standard deviation		2.83		3.20		3.58		3.57	3.15			(2) > (5)
	n		74		63		49		32	36			
TOTAL B	Average		2.07		2.08		1.96		1.66	1.94			
	Median		2.00		2.00		2.00		2.00	2.00	0.368		(1) = (2) = (3) = (4) = (5)
	Standard deviation		0.53		0.77		0.84		1.07	1.04			
	n		74		63		49		32	36			
Part 1 (total)	Average		10.84		8.78		7.88		6.56	6.22			
	Median		12.00		9.00		8.00		6.50	7.00	<0.001*		(1) > (2) = (3) = (4) = (5)
	Standard deviation		2.97		3.45		3.81		4.01	3.83			(2) > (4) = (5)
	n		74		63		49		32	36			
TOTAL C	Average		13.03		18.16		18.61		20.53	20.56			
	Median		12.50		21.00		21.00		21.00	21.00	<0.001*		(1) < (2) = (3) = (4) = (5)
	Standard deviation		5.78		5.16		4.89		1.48	1.50			
	n		74		63		49		32	36			
TOTAL D	Average		5.23		6.16		6.39		7.00	6.61			
	Median		6.00		7.00		7.00		7.00	7.00	<0.001*		(1) < (2) = (3) = (4) = (5)
	Standard deviation		2.30		2.06		1.68		0.44	1.20			
	n		74		63		49		32	36			
PART 2 (Total)	Average		18.26		24.32		25.00		27.53	27.17			
	Median		18.00		27.00		28.00		28.00	28.00	<0.001*		(1) < (2) = (3) = (4) = (5)
	Standard deviation		7.57		6.78		6.33		1.52	1.95			
	n		74		63		49		32	36			
TOTAL F	Average		2.91		4.25		4.90		5.34	5.69			
	Median		3.00		5.00		6.00		6.00	6.00	<0.001*		(1) < (2) = (3) = (4) = (5)
	Standard deviation		2.17		1.99		2.04		0.90	0.75			(2) < (5)
	n		74		63		49		32	36			
TOTAL G	Average		2.49		6.22		7.02		8.16	8.58			
	Median		1.00		7.00		9.00		9.00	9.00	<0.001*		(1) < (2) = (3) = (4) = (5)
	Standard deviation		2.90		3.07		3.21		1.71	1.56			(2) < (4) = (5)
	n		74		63		49		32	36			
TOTAL H	Average		10.54		20.21		23.14		27.97	33.17			(1) < (2) = (3) = (4) = (5)
	Median		6.50		21.00		29.00		30.00	36.00	<0.001*		(2) < (4) = (5)
	Standard deviation		10.79		11.58		13.17		8.82	5.64			(3) < (5)
	n		74		63		49		32	36			
TOTAL I	Average		3.88		8.44		9.94		12.16	13.00			(1) < (2) = (3) = (4) = (5)
	Median		2.00		9.00		12.00		13.00	14.00	<0.001*		(2) < (4) = (5)
	Standard deviation		4.36		5.92		4.60		2.19	1.47			(3) < (5)
	n		74		63		49		32	36			
TOTAL J	Average		0.66		1.56		2.18		2.31	3.00			
	Median		0.00		1.00		2.00		2.00	3.00	<0.001*		(1) < (2) = (3) = (4) = (5)
	Standard deviation		1.06		1.48		1.82		1.57	1.69			(2) < (5)
	n		74		63		49		32	36			
TOTAL K	Average	2.78		5.83		7.24		8.06		9.64			(1) < (2) = (3) = (4) = (5)
	Median	2.00		6.00		8.00		8.50		10.00		<0.001*	(2) < (4) = (5)
	Standard deviation	2.96		2.84		3.61		2.64		2.24			(3) < (5)
	n	74		63		49		32		36			
TOTAL M	Average	3.35		7.32		9.82		10.66		12.47			
	Median	2.00		8.00		11.00		11.00		13.00		<0.001*	(1) < (2) < (3) = (4) = (5)
	Standard deviation	3.38		4.28		4.68		3.34		2.92			(3) < (5)
	n	74		63		49		32		36			
TOTAL N	Average	3.66		11.92		17.67		21.81		25.36			
	Median	0.00		10.00		20.00		22.50		27.00		<0.001*	(1) < (2) < (3) = (4) = (5)
	Standard deviation	6.69		9.68		10.26		7.78		7.33			(3) < (5)
	n	74		63		49		32		36			
PART 3- (TOTAL)	Average	30.27		65.75		81.92		96.47		110.92			(1) < (2) = (3) = (4) = (5)
	Median	22.00		65.00		93.00		95.50		119.50		<0.001*	(2) < (4) = (5)
	Standard deviation	29.11		36.06		39.11		22.76		20.04			(3) < (5)
	n	74		63		49		32		36			
TOTAL LUI	Average	48.53		90.06		106.92		124.00		138.08			(1) < (2) = (3) = (4) = (5)
	Median	41.50		93.00		121.00		123.50		146.00		<0.001*	(2) < (4) = (5)
	Standard deviation	35.10		41.17		44.40		23.58		21.32			(3) < (5)
	n	74		63		49		32		36			

* Significative correlation

Caption: n = total number of participants per group; p = p value

Part 3 showed that the first 3 groups performed less than the groups of older children (4 and 5). G5 showed superior performance, especially in the subscales related to the use of sentences. This result can also be observed when analyzing the total LUI Score (Table 5).

Parental education also influenced the scores, according to the Kruskal-Wallis test, with Tukey's multiple comparisons (used when results were considered significant. Regarding mothers, there was an effect of years of schooling on Subscale C (p=0.011), Part 2 (p=0.017), Subscales F (p=0.024), G (p=0.010), and M (p=0.029), with children whose mothers had completed higher education obtaining significantly higher scores than those whose mothers had completed high school.

The results for fathers were similar to those for mothers. Except for Part 1, paternal education had an effect on all subscales and the Total LUI score. All children whose fathers had completed higher education obtained higher scores than those whose fathers had completed elementary and high school.

## DISCUSSION

The Language Use Inventory (LUI) provides a measure of a child’s language use in a reasonable and cost-effective manner^(24)^. The LUI has been used in research studies internationally, and has been translated and adapted into several languages. In Brazil, in the first stage of translation, back-translation was performed by the authors of this study^(34)^, and in the current study efforts were focused on demonstrating the reliability of the translated LUI-Brazilian Portuguese for the Brazilian population.

The reliability of the questionnaire was assessed for all parts of the LUI. Cronbach’s alpha analysis showed excellent reliability for all scored subscales, especially in Parts 2 and 3 that make up the LUI Total score. This result is in line with the first study conducted by Brocchi et al. ^(34)^, as well as all published cross-cultural adaptations^(8,28-33)^.

Looking at each subscale of the questionnaire, the results of the present research can be compared with those of the other mentioned adaptations and of the original LUI in English. Subscale B showed satisfactory internal consistency, which differs from the pilot research conducted by the authors as well as from other cross-cultural adaptations which observed low consistency in the same subscale^(8,28-30)^. In this study, as well as in the adaptation performed in the Chinese context^(32)^, this subscale showed an acceptable score. This satisfactory result was observed for subscales D and J as well. In the pilot study, the subscale D showed low internal consistency, and subscale J scored at the borderline of acceptable. In this study, consistency proved to be adequate. This study was carried out with the questionnaire already adapted into Brazilian Portuguese and with a much larger number of participants, which allowed for a more accurate analysis of the results.

After the reliability verification step, correlations were examined between the LUI’s subscales to further investigate the reliability of the adaptation process. This analysis showed the relationship between the subscales of Part 1 related to gestures, and how this relationship became significantly negative when compared to the subscales related to the production of words and sentences. The subscales in Parts 2 and 3, in turn, correlated strongly with each other, similar to the results of other adaptations into Chinese^(32)^, Norwegian^(31)^, and Canadian French^(29)^.

This study included children across the entire age range for which the LUI is designed, namely, children 18 to 47 months old. Given the larger number of participants in this study across this age range (compared to the translation process^(34)^) and the increase in performance to be expected during this time period, the sample was divided into five groups for a more detailed analysis. It was observed that the youngest group of children aged 18-24 months used more gestures in their communication, and as children grew older, gestural communication decreased and the use of words and sentences increased significantly. These results are in line with those of the original LUI^(8)^ and translations of the LUI^(28-33)^.

The effects of maternal and paternal education were observed on children’s scores on the subscales in Parts 2 and 3; i.e., parents with more years of schooling had a correlation with children’s production of words and sentences. The literature corroborates this finding since children’s language development may be influenced by environmental conditions, including the availability and richness of linguistic and cognitive stimulation provided by sensitive and responsive parents. A study^(35)^ observed the dyadic interactions of 2-year-old children. The results suggested that children whose parents had more years of schooling and income benefited from their parents’ verbal repertoires for vocabulary development and family stimulation in different contexts.

This responsiveness, however, can be influenced by individual child characteristics, such as sex^(36)^. In this study, except for the use of gestures, girls may use more words and phrases than boys; i.e., they scored significantly higher than boys on all subscales of Parts 2, 3 and Total Score. Authors^(8,16,37)^ observed superior performance of girls; Adaptations made for French^(19)^ and Mandarin^(32)^ observed significantly higher Total scores for girls in age groups between 18 and 30 months (first study) and between 18 and 23 months (second study). According to the authors, this difference decreased in the analyses of later age groups and became statistically insignificant. Researches^(31)^ noted significant results from girls only in subscales D and F (second study) but not in Total Scores. Italian study^(38)^ observed differences between the sexes in their study population. Girls performed higher than boys at younger ages but boys scored better at older ages (36 to 47 months).

The results described above have provided important analyses, showing that the questionnaire can be reproduced for the Brazilian population. The instrument is still being studied by the researchers and is currently in the process of being validated. Thus, the development of normative parameters and standard deviations is still under construction.

The COVID-19 pandemic and the restrictions that followed it, until the wide opening of services, led to a considerable delay in the survey schedule, even online, as families were overloaded with internet services, which also helped delay the sending of responses.

The data was collected in different environments, both face-to-face (in outpatient clinics, health centers, volunteer work) and online (it was disseminated on various social media (Whatsapp, Instagram, Facebook and Whatsapp groups of municipal schools that collaborated with the survey), to reach a broad population. The results showed that the majority of parents had completed high school and, in the online form, the parents who most volunteered to fill in the inventory were those with more years of schooling.

In the current phase of the research, the researchers intend to reach other education levels of the parents and age groups of the children, carry out detailed analyses, and construct normative scores.

## CONCLUSION

Based on these results, it was observed that the translated LUI- Brazilian Portuguese maintained high internal reliability similar to the original, and that the LUI presents high reliability for reproduction. An increase in the use of words and phrases with increasing age and a decrease in the use of gestures by older children was observed. This study analyzed the influence of sex and parents’ educational levels on the scores obtained in the questionnaire.

This questionnaire will provide Brazilian speech therapists with an efficient tool for evaluating and monitoring pragmatic language skills of typical and atypical children from 18 to 47 months of age.
